# An *Fgfr3*-activating mutation in immature murine osteoblasts affects the appendicular and craniofacial skeleton

**DOI:** 10.1242/dmm.048272

**Published:** 2021-04-23

**Authors:** Martin Biosse Duplan, Emilie Dambroise, Valentin Estibals, Joelle Veziers, Jérome Guicheux, Laurence Legeai-Mallet

**Affiliations:** 1 Laboratory of Molecular and Physiopathological Bases of Osteochondrodysplasia, INSERM UMR 1163, Imagine Institute, Paris 75015, France; 2 Université de Paris, Paris 75006, France; 3 Service de Médecine Bucco-Dentaire, Hôpital Bretonneau, AP-HP, Paris 75018, France; 4 Inserm, UMR 1229, RMeS – Regenerative Medicine and Skeleton, Université de Nantes, ONIRIS, Nantes, F-44042, France; 5 SC3M, SFR Santé F. Bonamy, FED 4203, UMS Inserm 016, CNRS 3556, Nantes F-44042, France; 6 CHU Nantes, PHU4 OTONN, Nantes, F-44093, France

**Keywords:** Chondrocyte, Osteoblast, Achondroplasia, FGFR3

## Abstract

Achondroplasia (ACH), the most common form of dwarfism, is caused by a missense mutation in the gene coding for fibroblast growth factor receptor 3 (*FGFR3*). The resulting increase in FGFR3 signaling perturbs the proliferation and differentiation of chondrocytes (CCs), alters the process of endochondral ossification and thus reduces bone elongation. Increased FGFR3 signaling in osteoblasts (OBs) might also contribute to bone anomalies in ACH. In the present study of a mouse model of ACH, we sought to determine whether FGFR3 overactivation in OBs leads to bone modifications. The model carries an *Fgfr3*-activating mutation (*Fgfr3^Y367C/+^*) that accurately mimics ACH; we targeted the mutation to either immature OBs and hypertrophic CCs or to mature OBs by using the *Osx-cre* and collagen 1α1 (2.3 kb *Col1a1*)*-cre* mouse strains, respectively. We observed that Fgfr3 activation in immature OBs and hypertrophic CCs (*Osx-Fgfr3*) not only perturbed the hypertrophic cells of the growth plate (thus affecting long bone growth) but also led to osteopenia and low cortical thickness in long bones in adult (3-month-old) mice but not growing (3-week-old) mice. Importantly, craniofacial membranous bone defects were present in the adult mice. In contrast, activation of Fgfr3 in mature OBs (*Col1-Fgfr3*) had very limited effects on skeletal shape, size and micro-architecture. *In vitro*, we observed that Fgfr3 activation in immature OBs was associated with low mineralization activity. In conclusion, immature OBs appear to be affected by Fgfr3 overactivation, which might contribute to the bone modifications observed in ACH independently of CCs.

## Abstract (precis)

**Editor's choice:** In a mouse model of achondroplasia, targeting an *Fgfr3*-activating mutation to immature osteoblasts led to osteopenia and induced craniofacial membranous bone defects.

## INTRODUCTION

Achondroplasia (ACH; the most common form of dwarfism in humans) is caused by a missense mutation in the gene coding for fibroblast growth factor receptor 3 (*FGFR3*); the mutation activates the receptor and its downstream signaling pathways (Horton et al., 2007; Rousseau et al., 1994). The disease is characterized by a number of clinical features, including short-limb dwarfism, lordosis, macrocephaly, frontal bossing, a small foramen magnum diameter and midface hypoplasia (Horton et al., 2007). *FGFR3* gain-of-function mutations are also associated with mild dwarfism (hypochondroplasia) and severe dwarfism (thanatophoric dysplasia I and II) (Ornitz and Legeai-Mallet, 2016).

It is well established that, in ACH, increased FGFR3 signaling in growth plate chondrocytes (CCs) perturbs their proliferation and differentiation, alters the endochondral ossification process and thus reduces bone elongation (Ornitz and Legeai-Mallet, 2016). Recently, it has been suggested that osteoblasts (OBs) might also be affected in ACH. Indeed, children with ACH show mandible hypoplasia, frontal bone defects and (in some cases) premature fusion of coronal sutures, suggesting that membranous ossification is disturbed by FGFR3 overactivation (Biosse Duplan et al., 2016; Di Rocco et al., 2014). Furthermore, adults with ACH have a low bone mineral density, which leads to osteopenia or osteoporosis (Matsushita et al., 2016). Muenke syndrome, the most common form of craniosynostosis, is also due to a single *FGFR3* gain-of-function mutation (Muenke et al., 1997).

The expression of FGFRs in CC and OB lineage cells is tightly regulated (Yu and Ornitz, 2007). During skeletal development, cells in mesenchymal condensation take on a chondrogenic profile and express FGFR3. In developing bone, FGFR3 is expressed in proliferating and prehypertrophic CCs and trabecular OBs. In mature bone, FGFR3 is detected in OBs lining the endosteum of cortical bone, along the surface of trabecular bone and in osteocytes (Xiao et al., 2004). With regard to cranial vault formation, FGFR1 and FGFR2 are expressed at the osteogenic front in proliferative and mature osteoblasts, respectively, whereas FGFR3 is expressed to a lesser extent during suture formation in immature and mature OBs located at the periphery of bone tissue (Rice et al., 2000). *In vitro*, OBs from calvaria express all the FGFRs (FGFR1 to FGFR4), whereas osteocytes only express FGFR1 and FGFR3 (Delezoide et al., 1998; Kyono et al., 2012; Murali et al., 2016; Paic et al., 2009).

The bone phenotype has been described in several mouse models carrying *Fgfr3* loss- or gain-of-function mutations. In an *Fgfr3* loss-of-function model, the absence of Fgfr3 leads to low bone mass and thinner cortical bone in adult mice (Eswarakumar and Schlessinger, 2007; Valverde-Franco et al., 2004), despite an increased OB count (Valverde-Franco et al., 2004). In the latter model, the absence of Fgfr3 increases early OB differentiation but interferes with the cells' activity, thus leading to defective mineralization. Surprisingly, Fgfr3 activation in mouse models of ACH (*Fgfr3^G369C/+^*, *Fgfr3^G380R/G380R^*, *Fgfr3^Y367C/+^*) also decreases bone formation and femoral bone mass and alters bone microarchitecture in growing and young adult mice (Lee et al., 2017; Mugniery et al., 2012; Su et al., 2010). In the *Fgfr3^Y367C/+^* mouse, targeting mature OBs (using the 2.3 kb *Col1a1-cre* strain) does not affect bone mass or microarchitecture in growing animals (Mugniery et al., 2012). The effects of this Cre strain in adult mice and the effects of Fgfr3 activation earlier in the course of OB differentiation have not previously been established.

Although it is clear that increased FGFR3 signaling leads to bone modifications in humans and mice, the respective contributions of altered FGFR3 signaling in specific cell types (namely CCs and OBs) to the bone phenotype have not been defined. Assessing the specific role of FGFR3 is important for finding a treatment for ACH; depending on the patient population (e.g. children versus adults), therapeutic strategies might conceivably target different cell types (Ornitz and Legeai-Mallet, 2016). In adults, targeting OBs may be more relevant than targeting CCs. In addition, understanding the effect of FGFR3 overactivation on the osteoblast is crucial for the development of treatments for craniosynostoses caused by *FGFR3* mutations. Characterization of the respective contributions of CCs and OBs is complex in mouse models, for two main reasons. First, several major genes [*Osx* (also known as *Sp7*), *Runx2* and *Col2a1*] are expressed in both CCs and OBs during the differentiation process. These include genes for which the promoters have been used to create Cre-expressing mouse strains (Elefteriou and Yang, 2011), meaning that both cell types are simultaneously targeted. Second, OBs originate from mesenchymal progenitors and follow different differentiation pathways. A substantial proportion of growth plate CCs transdifferentiate into OBs; this contributes to trabecular and cortical bone formation during endochondral ossification (Ono et al., 2014; Yang et al., 2014; Zhou et al., 2014). It is not known to what extent (if any) the differentiation and activity of OBs derived from disturbed growth plate CCs are modified in ACH.

In the present study, we sought to determine whether Fgfr3 overactivation in OBs leads to bone modifications in a mouse model of ACH. We took advantage of a model carrying an *Fgfr3*-activating mutation (*Fgfr3^Y367C/+^*) that accurately mimics ACH (Pannier et al., 2009), and targeted the mutation to either immature OBs and hypertrophic CCs or to mature OBs by using the *Osx-cre* and 2.3 kb *Col1a1-cre* mouse strains, respectively. We found that Fgfr3 activation in immature OBs and hypertrophic CCs not only perturbed hypertrophic cells in the growth plate (thus affecting long bone growth) but also led to osteopenia in adult (3-month-old) mice but not in growing (3-week-old mice). Importantly, growing mice displayed a defective frontal bone formation. *In vitro*, we observed that Fgfr3 activation in immature OBs isolated from calvaria reduced the cells' mineralization activity. In contrast, activation of Fgfr3 in mature OBs had very limited effects on skeletal shape, size and micro-architecture.

## RESULTS

### Fgfr3 activation in immature OBs and hypertrophic CCs leads to dwarfism and growth plate anomalies

**Fig. 1.**
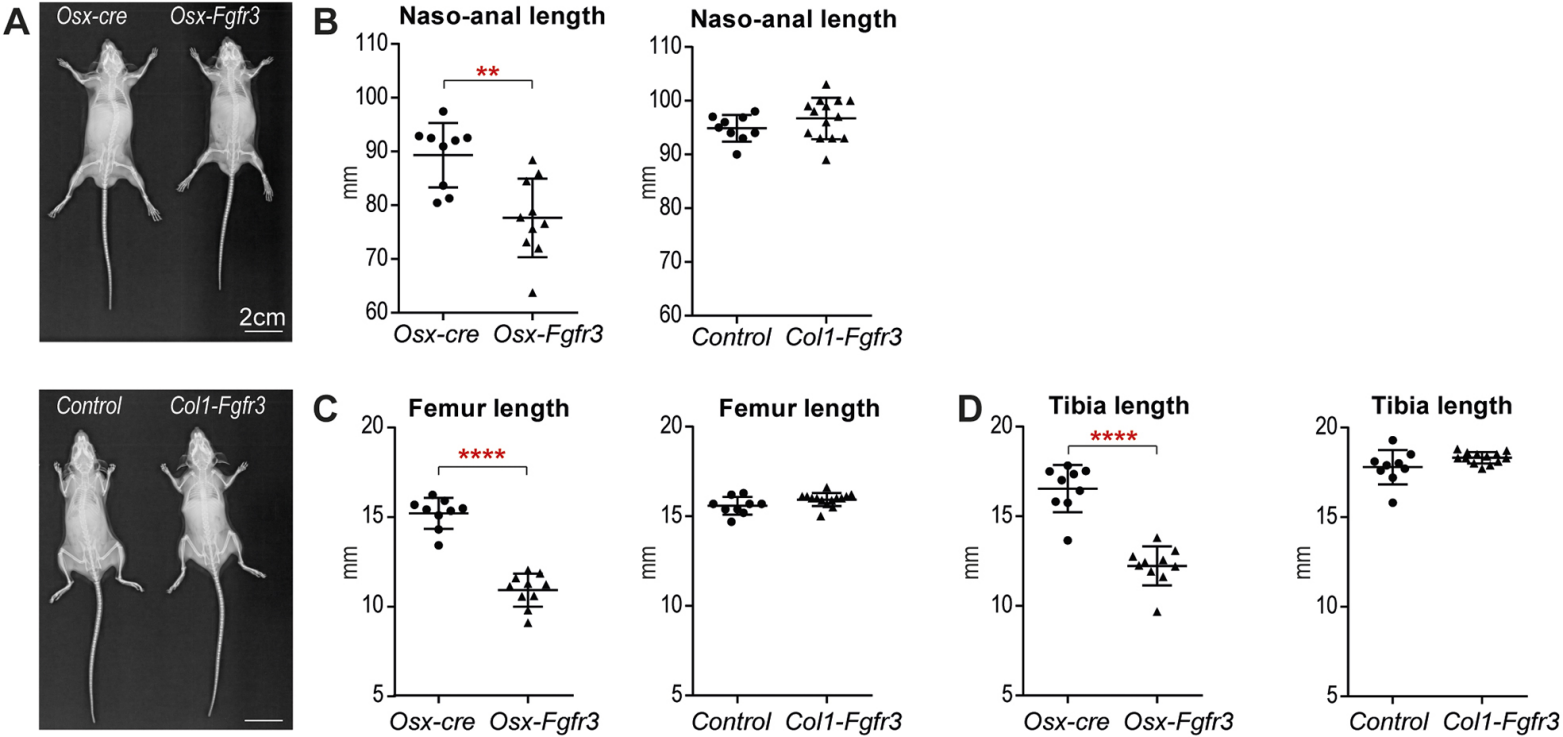
**Fgfr3 activation in immature OBs and hypertrophic CCs leads to mild dwarfism.** (A) Representative X-rays of 3-month-old *Osx^cre/+^*-*Fgfr3^Y367C/+^* mice (referred to here as *Osx-Fgfr3*), *Osx^cre/+^* littermates (*Osx-cre*), *Col1a1^cre/+^-Fgfr3^Y367C/+^* mice (*Col1-Fgfr3*) and wild-type or *Col1a1^cre/+^* littermates (controls). (B) Mean±s.d. naso-anal distance for 3-month-old *Osx-Fgfr3*, *Osx-cre*, *Col1-Fgfr3* and control mice. (C) Mean±s.d. length of the left femur in 3-month-old *Osx-Fgfr3*, *Osx-cre*, *Col1-Fgfr3* and control mice. (D) Mean±s.d. length of the left tibia in 3-month-old *Osx-Fgfr3*, *Osx-cre*, *Col1-Fgfr3* and control mice. Each point represents an individual mouse (*n*≥8 for each genotype); **P≤0.01 and ****P≤0.0001 compared with the appropriate control group (unpaired Student's *t*-test).

The groups of *Osx-Fgfr3* (*Osx^cre/+^*-*Fgfr3^Y367C^*^/+^) and *Col1-Fgfr3* (*Col1a1^cre/+^-Fgfr3^Y367C^*^/+^) mice were born with the expected Mendelian ratio. At 3 months of age, imaging showed that bone growth was impaired in *Osx-Fgfr3* mice, relative to *Osx-cre* mice (Fig. 1A). The *Osx-Fgfr3* mice had a shorter naso-anal distance (by 13.1% versus the comparator mice; *P*≤0.005), a shorter femur (by 28.3%; *P*≤0.001) and a shorter tibia (by 26.1%; *P*≤0.001) (Fig. 1B-D). In contrast, *Col1-Fgfr3* mice were indistinguishable from controls (Fig. 1B-D).

**Fig. 2.**
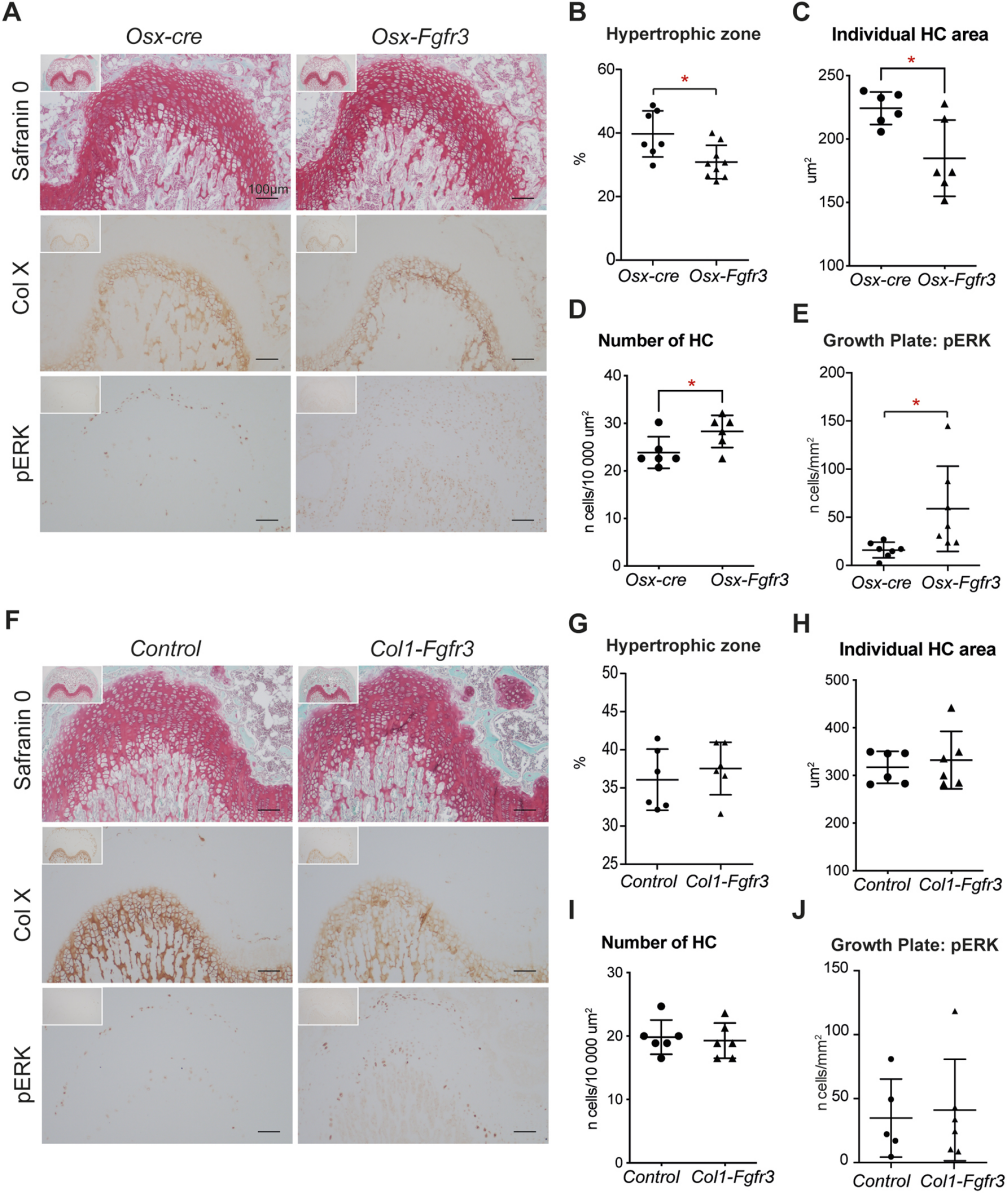
**Fgfr3 activation in immature OBs and hypertrophic CCs leads to growth plate anomalies.** (A) Representative histological sections of femurs of 3-week-old *Osx-Fgfr3* and *Osx-cre* mice, stained with Safranin O (to identify the growth plate cartilage; top row) and immunolabeled for collagen type X (to identify hypertrophic CCs; middle row) and pERK1/2 (bottom row). (B) Quantification of the size of the hypertrophic zone in the growth plate. (C) Mean area of individual hypertrophic chondrocytes (HCs) of the hypertrophic zone in the growth plate. (D) Quantification of the number of HCs per surface in the hypertrophic zone of the growth plate. (E) Quantification of pERK1/2-positive cells in the growth plate. (F) Representative histological sections of femurs of 3-week-old *Col1-Fgfr3* and control mice stained with Safranin O (to identify the growth plate cartilage; top row) and immunolabeled for collagen type X (to identify hypertrophic CCs; middle row) and pERK1/2 (bottom row). (G) Quantification of the size of the hypertrophic zone in the growth plate. (H) Mean area of individual HCs of the hypertrophic zone in the growth plate. (I) Quantification of number of HCs per surface in the hypertrophic zone of the growth plate. (J) Quantification of pERK1/2-positive cells in the growth plate. Each point represents an individual mouse (*n*≥6 for each genotype); *P≤0.05 compared with the appropriate control group (unpaired Student's *t*-test).

The mild dwarfism observed in *Osx-Fgfr3* mice prompted us to look at the growth plate of long bones in growing and adult animals. Following Safranin O staining and collagen X immunostaining (to identify cartilage and hypertrophic CCs), we observed that the ratio between the hypertrophic zone and growth plate cartilage was abnormally low in *Osx-Fgfr3* mice (by 22.1%; *P*≤0.05) at 3 weeks of age (Fig. 2A,B), suggesting a chondrocyte differentiation defect. This defect can be associated with a modification of the area of individual hypertrophic chondrocytes (HCs). Indeed, we found that the mean area of HCs was smaller in *Osx-Fgfr3* compared to *Osx-cre* mice (−17.5%; *P*<0.05) (Fig. 2C). Accordingly, there was a small increase in the number of HCs per surface in *Osx-Fgfr3* compared to *Osx-cre* mice (+18.4%; *P*<0.05) (Fig. 2D). The reduced size of HCs in the *Osx-Ffgr3* mutants suggested a defect in chondrocyte differentiation induced by increased Fgfr3 signaling, as observed in other models (Biosse Duplan et al., 2016; Komla-Ebri et al., 2016). To measure whether increased Fgfr3 signaling was indeed present in these cells, we studied the Mapkinase pathway (Komla-Ebri et al., 2016). In *Osx-Fgfr3* mutants, phosphorylation of ERK1/2 (pERK1/2) was increased in the growth plate (by 239%; *P*≤0.05), mainly in HCs, compared to *Osx-cre* mice (Fig. 2A,E), reflecting increased Fgfr3 signaling in these mice.

In contrast, the proliferation of the CCs in the whole growth plate was not modified in *Osx-Fgfr3* mice (Fig. S1A,B); this was expected, because the *Osx-cre* mouse strain does not target proliferative CCs (Elefteriou and Yang, 2011). The proliferation of OBs in the bone marrow was not altered either (Fig. S1A,B). The abnormally low ratio between the hypertrophic zone and growth plate cartilage persisted at 3 months of age (18.5% lower; *P*≤0.05) (Fig. S2A,B). No such defects were observed in *Col1-Fgfr3* mice at 3 weeks or 3 months of age (Fig. 2F-I; Fig. S2C,D). As expected, in the *Col1-Fgfr3* mutants, we did not detect abnormal expression of pERK1/2 owing to increased Fgfr3 signaling in the growth plate (Fig. 2F,J).

### Fgfr3 activation in immature OBs leads to osteopenia

**Fig. 3.**
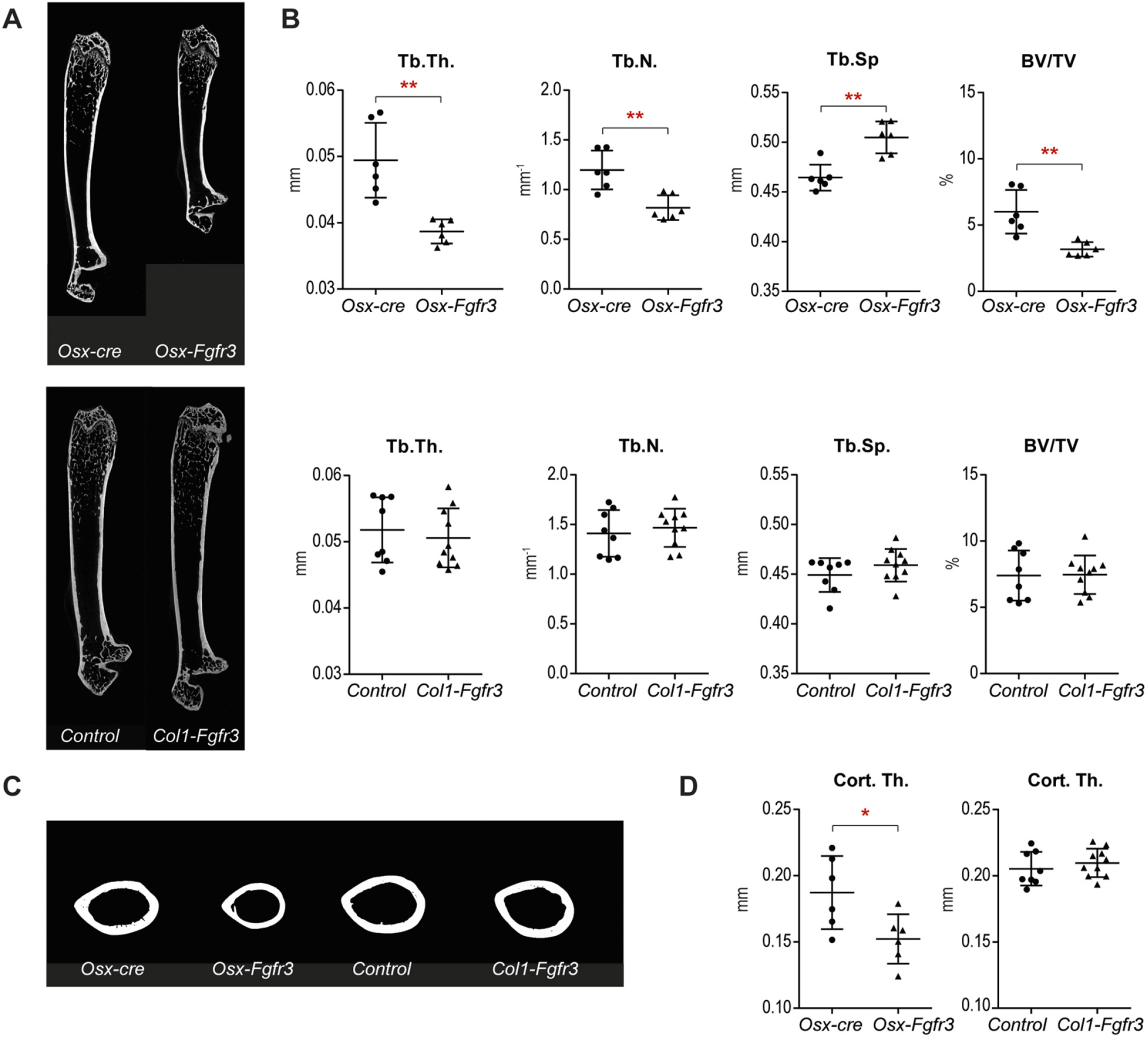
**Fgfr3 activation in immature OBs leads to osteopenia in adult animals.** (A) Representative two-dimensional (2D) sections of the right femurs in 3-month-old *Osx-Fgfr3*, *Osx-cre*, *Col1-Fgfr3* and control mice, generated from the µCT data. (B) Trabecular micro-architecture of the right femurs in 3-month-old *Osx-Fgfr3*, *Osx-cre*, *Col1-Fgfr3* and control mice, as assessed with µCT. (C) Representative 2D sections of the right femurs in 3-month-old *Osx-Fgfr3*, *Osx-cre*, *Col1-Fgfr3* and control mice sectioned at mid-shaft, generated from the µCT data. (D) Cortical micro- and macro-architecture of the right femurs in 3-month-old *Osx-Fgfr3*, *Osx-cre*, *Col1-Fgfr3* and controls, as assessed with µCT. BV/TV, bone volume divided by total volume; Cort.Th., cortical thickness; Tb.N, trabecular number; Tb.Sp., trabecular separation; Tb.Th, trabecular thickness. Each point represents an individual mouse (*n*≥6 for each genotype); *P≤0.05 and **P≤0.01 compared with the appropriate control group (unpaired Student's *t*-test).

To analyze the effect of *Fgfr3* overactivation in OBs on bone mass and microarchitecture, we used micro-computed tomography (µCT) to image the femurs of 3-week-old and 3-month-old mice. We did not observe a difference in the volume or architecture of trabecular bone and cortex in 3-week-old *Osx-Fgfr3* mice compared with *Osx-cre* mice (Fig. S3). In contrast, marked osteopenia was present in adult (3-month-old) *Osx-Fgfr3* mice compared with *Osx-cre* mice (Fig. 3A,B). The trabecular thickness and trabecular number were lower than in controls [by 23% (*P*≤0.005) and 34% (*P*≤0.01), respectively], whereas trabecular separation was higher (+10%; *P*≤0.01). This resulted in a low bone volume (by 46%; *P*≤0.005). Cortical thickness was also abnormally low in adult *Osx-Fgfr3* mice (by 19%; *P*≤0.05) (Fig. 3C,D). Adult *Col1-Fgfr3* mice did not show any significant differences in bone mass, bone microarchitecture or cortical thickness (Fig. 3A-D).

### Fgfr3 activation in immature OBs and hypertrophic CCs affects craniofacial growth and induces cranial base anomalies

**Fig. 4.**
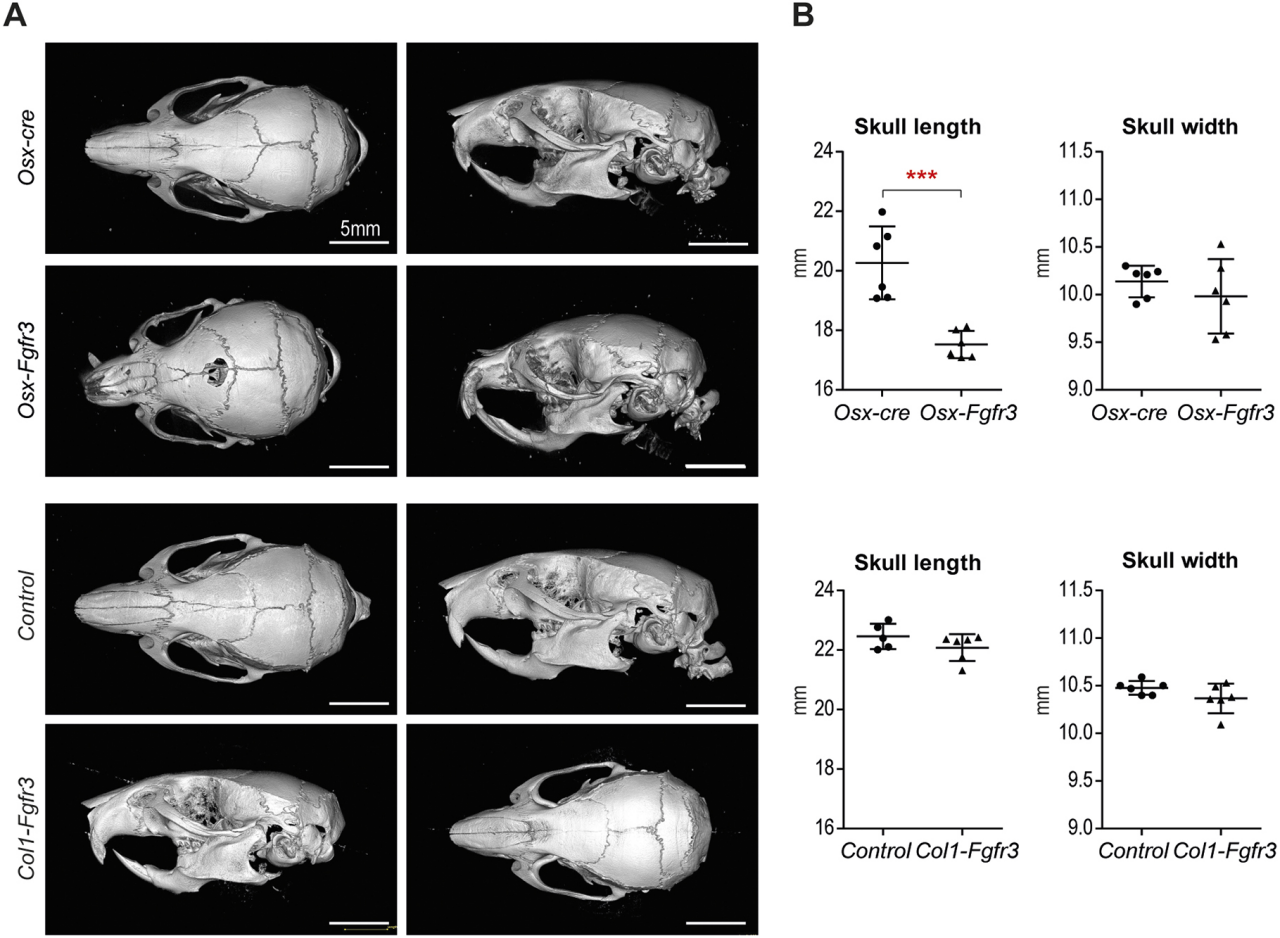
**Fgfr3 activation in immature OBs and hypertrophic CCs affects skull growth.** (A) Representative 3D reconstructions of the skull in 3-month-old *Osx-Fgfr3*, *Osx-cre*, *Col1-Fgfr3* and control mice. (B) Quantification of the length and width (measured on µCT) of the skull in 3-month-old *Osx-Fgfr3*, *Osx-cre*, *Col1-Fgfr3* and control mice. Each point represents an individual mouse (*n*≥5 for each genotype); ***P≤0.01 compared with the appropriate control group (unpaired Student's *t*-test).

Bone growth of the skull depends on both endochondral and membranous ossification. Elongation of the cranial base relies on synchondroses – cartilaginous growth centers that separate the different bones of the base formed by endochondral ossification. In *Osx-Fgfr3* adult mutants, the overall shape of the skull was modified, with a shorter skull (by 14%; *P*≤0.001) (Fig. 4A,B).

**Fig. 5.**
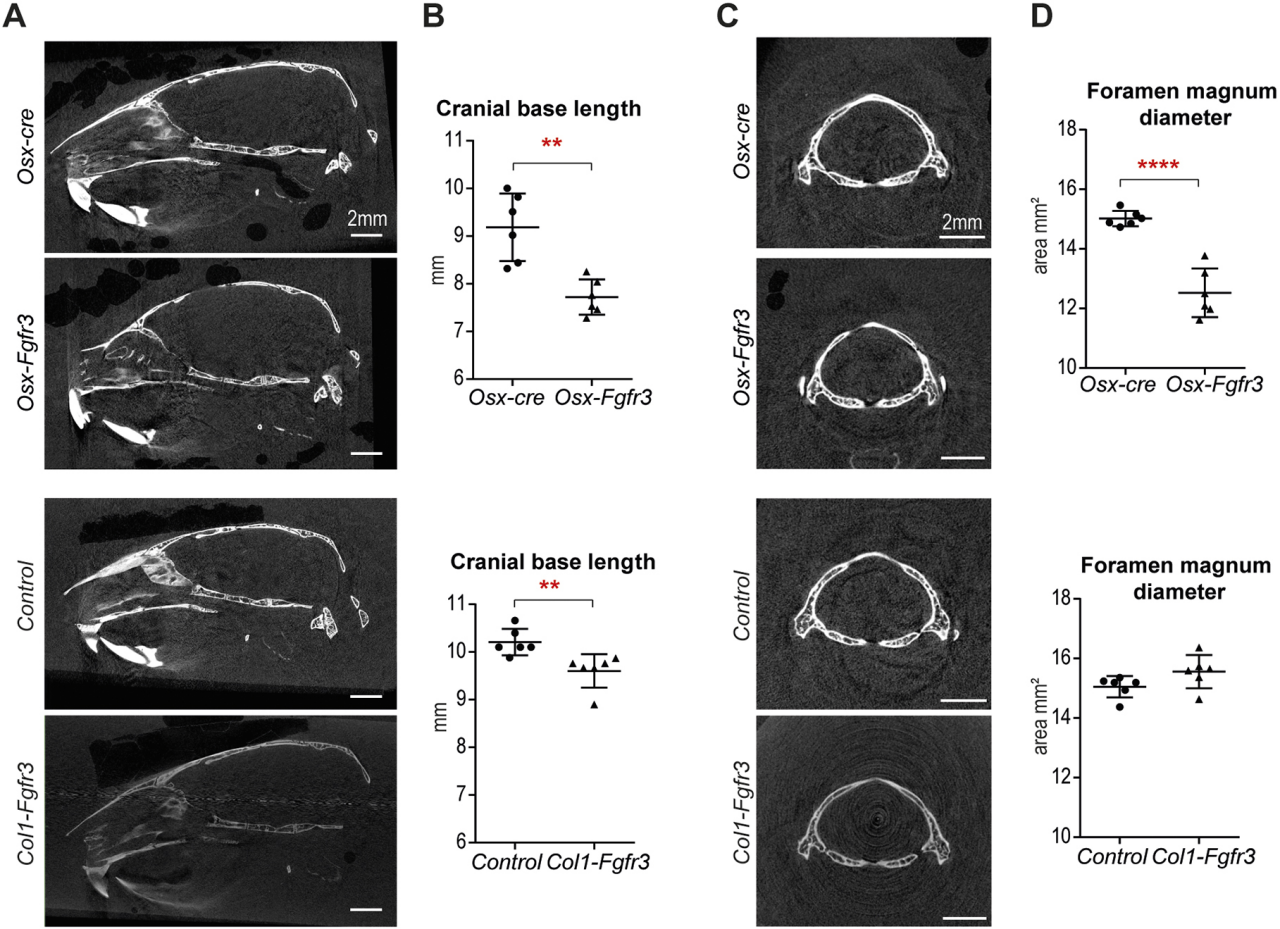
**Fgfr3 activation in immature OBs and hypertrophic CCs affects the cranial base.** (A) Representative 2D midline sagittal sections of the skull in 3-month-old *Osx-Fgfr3*, *Osx-cre*, *Col1-Fgfr3* and control mice, generated from the µCT data. (B) Mean±s.d. length of the cranial base in 3-month-old *Osx-Fgfr3*, *Osx-cre*, *Col1-Fgfr3* and control mice. (C) Representative 2D axial sections of the foramen magnum in 3-month-old *Osx-Fgfr3*, *Osx-cre*, *Col1-Fgfr3* and control mice, generated from the µCT data. (D) Mean±s.d. diameter of the foramen magnum in 3-month-old *Osx-Fgfr3*, *Osx-cre*, *Col1-Fgfr3* and control mice. Each point represents an individual mouse (*n*≥5 for each genotype); **P≤0.01 and ****P≤0.0001 compared with the appropriate control group (unpaired Student's *t*-test).

The cranial base and the diameter of the foramen magnum were also smaller [by 16% (*P*≤0.005) and 17% (*P*≤0.001), respectively] in these mice (Fig. 5A-D). We did not observe premature fusion of the synchondroses (Fig. 5A).

The length of the skull was not modified in *Col1-Fgfr3* mutants (Fig. 4A,B), but we did observe a slightly shorter cranial base (by 6%; *P*≤0.01) (Fig. 5A,B). More specifically, the presphenoid was shorter (by 15%; *P*≤0.005) in *Col1-Fgfr3* mutants than in controls. The two other bones of the cranial base (the sphenoid and occipital) were not affected. The diameter of the foramen magnum did not differ (Fig. 5C,D). Again, we did not observe premature fusion of synchondroses (Fig. 5A).

### Fgfr3 activation in immature OBs causes defective membranous ossification of the skull

We next analyzed the effect of Fgfr3 activation on membranous ossification in the cranial vault, to which CCs do not contribute. *Osx-Fgfr3* adult mutants frequently showed a skewed snout and defects in frontal and nasal bones (Fig. 4A). The frontal bone defects were related to weak growth of the bone edges in the sagittal direction, resulting in a large hole. The nasal bone defects consisted of small holes in the bones. Some of the *Osx-cre* mice also showed this type of defect, albeit less frequently than the *Osx-Fgfr3* mice (Table 1). On 3D reconstructions, the average area of the frontal bone defect was much greater in *Osx-Fgfr3* mice than in *Osx-cre* mice. The membranous defects were absent in *Col1-Fgfr3* mutants (Fig. 4A and Table 1).

**Table 1.**
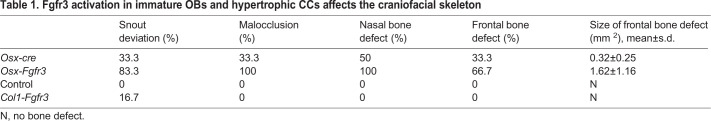
**Fgfr3 activation in immature OBs and hypertrophic CCs affects the craniofacial skeleton**

### Fgfr3 activation in immature OBs modifies OB activity

**Fig. 6.**
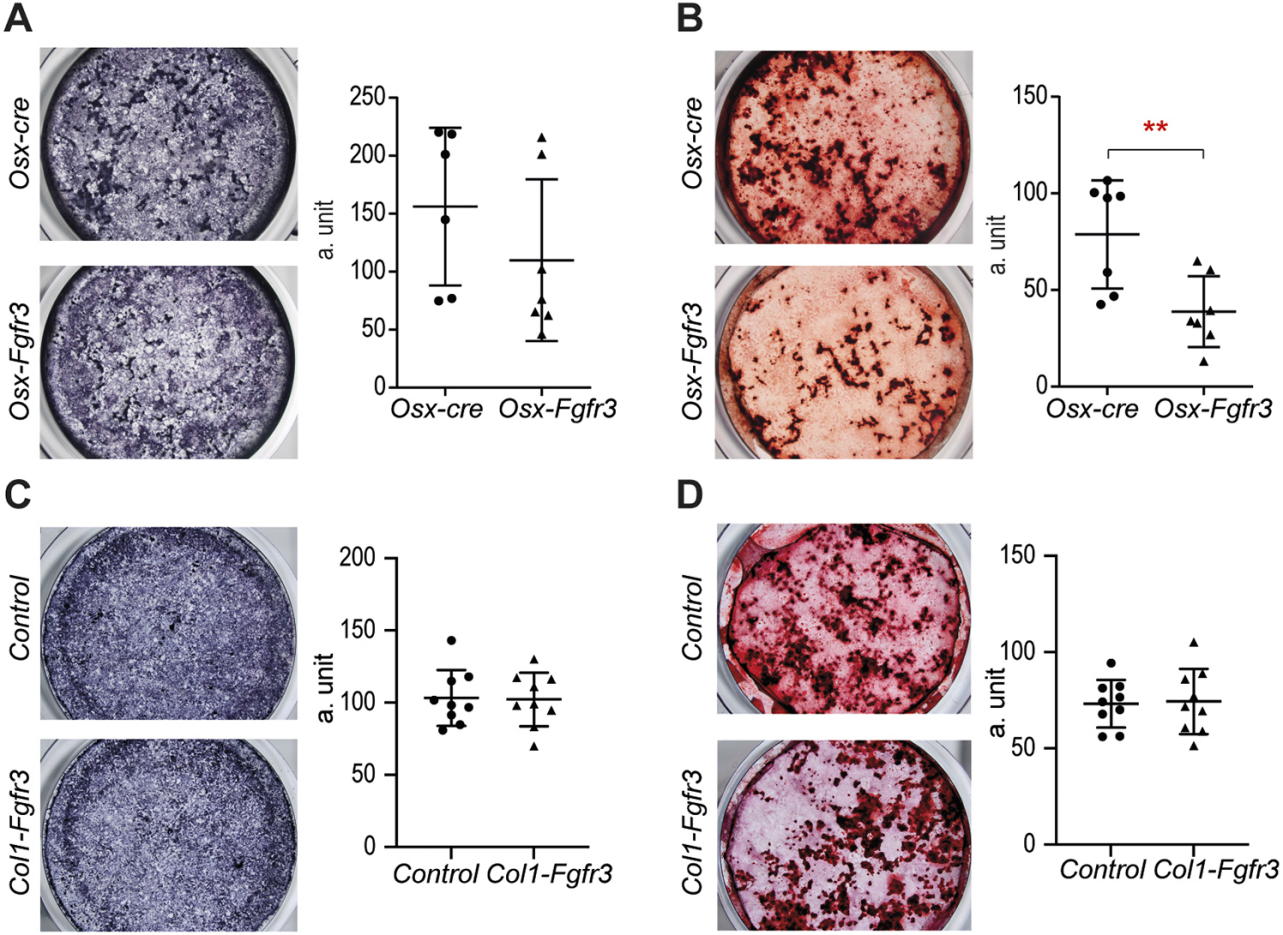
**Fgfr3 activation in immature OBs impairs OB activity.** (A) Representative photographs of OBs from *Osx-Fgfr3* and *Osx-cre* mice stained for ALP after 7 days of culture; mean±s.d. intensity of ALP staining of OBs from *Osx-Fgfr3* and *Osx-cre* mice after 7 days of culture. (B) Representative photographs of OBs from *Osx-Fgfr3* and *Osx-cre* mice stained with Alizarin Red after 21 days of culture; mean±s.d. intensity of Alizarin Red staining of OBs from *Osx-Fgfr3* and *Osx-cre* mice after 21 days of culture. (C) Representative photographs of OBs from *Col1-Fgfr3* and control mice stained for ALP after 7 days of culture; mean±s.d. intensity of ALP staining of OBs *Col1-Fgfr3* and control mice after 7 days of culture. (D) Representative photographs of OBs from *Col1-Fgfr3* and control mice stained with Alizarin Red after 21 days of culture; mean±s.d. intensity of Alizarin Red staining of OBs from *Col1-Fgfr3* and control mice after 21 days of culture. a. unit, arbitrary unit. Each point represents an individual mouse (*n*≥6 for each genotype); **P≤0.01 compared with the appropriate control group (unpaired Student's *t*-test).

To determine whether the osteopenia and cranial vault defects observed in adult *Osx-Fgfr3* mice were caused by impaired OB proliferation, differentiation and/or activity, we cultured OBs generated from calvaria of *Osx-cre* and *Osx-Fgfr3* mice *in vitro*. A live-cell analysis of the cell number and viability using IncuCyte technology did not show any differences between OBs from *Osx-cre* and *Osx-Fgfr3* mice (Fig. S4). Likewise, we did not observe differences in OB differentiation [as shown by alkaline phosphatase (ALP) staining] between the genotypes (Fig. 6A). However, we found that OBs from *Osx-Fgfr3* mice formed fewer mineralization nodules (by 51%) after 3 weeks of culture than OBs from *Osx-cre* mice (*P*<0.01; Fig. 6B). OBs from *Col1-Fgfr3* mice did not show any significant differences in OB differentiation and activity (Fig. 6C,D).

## DISCUSSION

Although it is well established that *FGFR3*-activating mutations in chondrodysplasia primarily affect CCs and that changes in the proliferation and differentiation of these cells are responsible for the defective elongation of long bones (Ornitz and Legeai-Mallet, 2016), several observations in humans and in animal models suggest that OBs are also affected by elevated FGFR3 signaling. Adults with ACH or hypochondroplasia (two of the most common types of non-lethal chondrodysplasia) have a low spinal bone mineral density (Matsushita et al., 2016) and frequently suffer from osteopenia and osteoporosis (Arita et al., 2013). Furthermore, several cases of premature fusion of cranial sutures (a process that involves OBs but not CCs) (Georgoulis et al., 2011; Bessenyei et al., 2013; Albino et al., 2015) and delayed membranous ossification of the cranial vault have been reported in children with ACH (Di Rocco et al., 2014). In a mouse model of ACH, growing animals displayed severe osteopenia (Mugniery et al., 2012), partial premature fusion of sutures and membranous ossification defects (Di Rocco et al., 2014). In order to design innovative therapeutic strategies for targeting specific cell types and correcting the aforementioned defects, it is essential to determine the contribution of OBs to long bone modifications in ACH. To determine whether OBs are indeed affected by FGFR3 overactivation during membranous and endochondral ossification and bone remodeling, we used a Cre/lox approach to target the *Fgfr3^Y367C^* mutation to OBs in mice.

Our study results showed that targeting the *Fgfr3^Y367C^* mutation to cells expressing the Cre recombinase under the control of the *Osx* promoter impairs bone development and leads to short stature. This Cre strain targets not only immature OBs but also a subset of CCs – mostly hypertrophic CCs (Chen et al., 2014; Zhou et al., 2010). The expression of the mutant *Fgfr3* allele and elevated level of pERK1/2, impairing the differentiation in these CCs, might explain why the hypertrophic zone was smaller in the growth plate of *Osx-Fgfr3* mice and why longitudinal growth of the appendicular skeleton was defective. This finding was reinforced by the observation that the proliferation of CCs in the proliferative zone (where CCs do not express Osx) was not modified in *Osx-Fgfr3* mice. The defective CC differentiation observed in the hypertrophic zone in the growth plate of *Osx-Fgfr3* mice is reminiscent (although much milder) of that observed when the same mutation is expressed either ubiquitously or in CCs and osteochondroprogenitors (Mugniery et al., 2012). The dwarfism observed in *Osx-Fgfr3* mice is also much less severe than in these two strains of mice, owing to the lack of Fgfr3 expression in proliferative CCs in the present model. In contrast, we did not observe obvious growth plate and bone anomalies when using the 2.3 kb *Col1a1-cre* strain, which does not target CCs. The fact that *Col1-Fgfr3* mice had a normal body size indicates that FGFR3 overactivation in mature OBs does not affect long bone growth.

The cranial base and the foramen magnum diameter were also affected in *Osx-Fgfr3* mice, and their skull was brachycephalic. Elongation of the cranial base during growth is critical for attaining a normal skull length and a normal foramen magnum diameter. The process is primarily controlled by synchondroses that separate the different bones of the cranial base. The synchondroses and the CCs they contain are organized in much the same way as the growth plates of long bones (Wei et al., 2016), and the *Fgfr3^Y367C^* mutation affects the two sites in a similar manner (Di Rocco et al., 2014). It is therefore reasonable to assume that the reduced length of the cranial base in *Osx-Fgfr3* mutants resulted from the defective differentiation of CCs in the synchondroses, as observed in another model of Fgfr3 activation (Matsushita et al., 2008). Unexpectedly, we observed a small reduction in the length of the cranial base in *Col1-Fgfr3* mutants, even though the overall sagittal length of the skull and the foramen magnum diameter were not affected. This observation suggests that Fgfr3 overactivation in OBs alone, independently of CCs, could also affect bone elongation in the cranial base.

The exact role of FGFR3 during bone remodeling has not been established. Total deletion of *Fgfr3* results in osteopenia and thinner cortical bone in adult mice (Eswarakumar and Schlessinger, 2007; Valverde-Franco et al., 2004), and the absence of Fgfr3 increases early OB differentiation but interferes with the cells' normal activity, thus leading to defective mineralization. Specific deletion of *Fgfr3* in CCs is associated with greater bone formation and thus greater bone mass (Wen et al., 2016). Fgfr3 overactivation is also associated with low bone formation, low bone mass and altered bone micro-architecture in mice (Lee et al., 2017; Mugniery et al., 2012; Su et al., 2010). Along with defective long bone elongation, adult *Osx-Fgfr3* mice also displayed marked osteopenia, as shown by the low trabecular bone volume and low cortical thickness. When Fgfr3 overactivation was present later in OB differentiation, the bone mass was not affected. Given the influence of growth plate disorganization on trabecula formation and trabecular micro-architecture, it is difficult to establish the contribution of OBs and bone remodeling to this phenotype in adult mice with *Fgfr3* mutations. The primary and secondary spongiosa are severely affected in growing *Fgfr3^Y367C/+^* mice when the mutation is expressed ubiquitously and targets all CCs (Mugniery et al., 2012). In contrast, we did not observe osteopenia in growing *Osx-Fgfr3* mice at the age of 3 weeks, suggesting that the low bone mass observed at 3 months is independent of growth plate anomalies and is due (at least in part) to defective bone remodeling, rather than poor bone mass gain.

Because a large number of OBs are derived from growth plate CCs (Tsang and Cheah, 2019; Yang et al., 2014), we cannot rule out the possibility that OBs in *Osx-Fgfr3* adult mice are defective during bone formation and remodeling because they were targeted by the mutation when they were hypertrophic CCs. Transdifferentiated CCs from the growth plate might contribute less to cortical bone growth than to trabecular bone growth (Tsang and Cheah, 2019; Yee et al., 2018). In this respect, it is important to note that the cortical thickness was low in adult *Osx-Fgr3* mice.

Our observation of skull defects in adult *Osx-Fgfr3* mice demonstrates that Fgfr3 overactivation in OBs affects bone formation. Several bones formed by membranous ossification (i.e. in the absence of a cartilage template, such as nasal and frontal bones) were affected by Fgfr3 overactivation in *Osx-Fgfr3* mutants. Accordingly, when the same *Fgfr3* mutation that we used here was targeted to HCs alone, using the *ColX-cre* strain (Gebhard et al., 2008), the skull defects were absent (M.B.D., unpublished data). These skull defects were not systematically observed in *Osx-Fgfr3* mutants, and an incompletely penetrant skull phenotype has also been observed in another mouse model of Fgfr3 overactivation (Twigg et al., 2009). Furthermore, it has been reported that the *Osx-cre* transgene alone causes craniofacial defects (Huang and Olsen, 2015; Wang et al., 2015). It was therefore important to use appropriate controls (e.g. *Osx-cre*) in our study and to quantify the frequency of skull bone defects. Hence, we found that these membranous defects were more frequent and more severe in mutants than in controls, and so they likely resulted from Fgfr3 overactivation. This is in line with previous observations of membranous ossification defects in the skulls of mice with ubiquitous Fgfr3 overactivation and in children with ACH (Di Rocco et al., 2014). The *Osx-Fgfr3* mice often had a shortened, skewed snout, resulting in dental malocclusion and perturbed incisor wear. The mice had abnormally simple premaxillary sutures, rather than normal, interdigitated sutures. A similar phenotype is seen in the mouse model of Muenke syndrome – a form of craniosynostosis caused by FGFR3 overactivation (Twigg et al., 2009). In agreement with an effect of FGFR3 activation on OBs, we observed *in vitro* that the mineralization of calvaria OBs isolated from *Osx-Fgfr3* mice was impaired, even though there were no obvious differences in proliferation (*in vitro* and *in vivo*) and differentiation. These results do not rule out a role of Fgfr3 activation in the regulation of osteoprogenitor proliferation (Kawane et al., 2018). Our data on the likely role of Fgfr3 in OB homeostasis during calvarium development are in line with our recent studies of an *fgfr3* loss-of-function zebrafish model, which displayed major cranial vault defects, and impairments in immature OB expansion and differentiation (Dambroise et al., 2020).

Given the osteopenia, low cortical thickness and membranous craniofacial defects observed in adult *Osx-Fgfr3* mice and the impaired mineralization by OBs observed *in vitro*, we conclude that OBs are indeed affected by Fgfr3 overactivation and contribute to bone modifications independently of CCs. Our results further characterize the role of FGFR3 in osteogenesis, in which FGFR3 overactivation during early-stage OB differentiation impairs this cell lineage's activity. Future pharmacological treatments of ACH should therefore be carefully analyzed for their effects on both CC and OB differentiation and activity in growing and adult patients.

## MATERIALS AND METHODS

### Study approval

All experimental procedures and protocols were approved by the French national animal care and use committee (APAFIS#24826-2018080216094268 v5), in compliance with the EU Directive 2010/63/EU for animals. Experiments were carried out according to the European Convention for the Protection of Vertebrates Used for Scientific Purposes.

### Mouse strains

We used the *Osx-cre* (Rodda and McMahon, 2006) and *Col1a1-cre* (Dacquin et al., 2002) mouse strains to generate mouse models that expressed the Y367C mutation (*Fgfr3^neoY367C^*) (Pannier et al., 2009) in either immature OBs and hypertrophic CCs (*Osx-Fgfr3*) or mature OBs (*Col1-Fgfr3*). We crossed *Fgfr3*^neoY367C/+^ mice with hemizygous transgenic *Osx-cre* or 2.3 kb *Col1a1-cre* mice to excise the NEO sequence from the germline and thus generated *Osx^cre/+^*-*Fgfr3^Y367C/+^*, *Col1a1^cre/+^-Fgfr3^Y367C/+^* mice. In order to take account of the bone phenotype of the *Osx-cre* transgenic mouse, *Osx-Fgfr3* mice were compared with *Osx^cre/+^* littermates. *Col1-Fgfr3* mice were compared with either wild-type or *Col1a1^cre/+^* littermates. Cre activity of the *Col1a1-cre* line was assessed using double-fluorescent Cre reporter mice (mT/mG) (Muzumdar et al., 2007), as well as genomic PCR of wild-type and mutant alleles in calvarial osteoblasts cultures isolated from *Col1-Fgfr3* mice and controls. All experiments were performed on 3-week-old or 3-month-old male mice. At least six mice from each group were analyzed. All mice had a C57BL/6 background and were genotyped using a PCR assay of a sample of tail DNA, as described previously (Pannier et al., 2009).

### Histology and immunohistochemistry

The femurs and cranial base from *Osx-cre*, *Osx-Fgfr3* and *Col1-Fgfr3* mice and their control littermates were fixed in 4% paraformaldehyde at 4°C for 24 h, washed in PBS, decalcified in 0.5 M EDTA (pH 8.0) for a week or a month (depending on mouse age), dehydrated in graded ethanol solutions, cleared in xylene and embedded in paraffin. Five-micrometer sections were cut, stained with Safranin O and immunohistochemically stained using standard protocols. Antibodies against collagen type X (1:50; BIOCYC, Luckenwalde, Germany), pERK1/2 (1:500; Cell Signaling Technology, USA) and Ki67 (also known as Mki67; 1:3000; Abcam, Cambridge, MA, USA) and the Dako Envision kit (Dako North America, CA, USA) were used. Images were acquired with a PD70-IX2-UCB microscope (Olympus, Tokyo, Japan) and analyzed using ImageJ software (National Institutes of Health, Bethesda, MD, USA). Mean areas of individual HCs and number of HCs were measured from collagen X-labeled sections, within multiple 8500 µm^2^ regions of interest on sections immunostained for collagen type X.

### X-rays and µCT analyses

*Osx-cre*, *Osx-Fgfr3* and *Col1-Fgfr3* mice and their control littermates were sacrificed at 3 weeks or 3 months of age. The mice were measured and whole-body X-rays were performed with a digital cabinet X-ray system (LX-60, Faxitron, Tucson, AZ, USA). The anatomic features on the X-rays were measured with ImageJ software. The femurs and whole heads were dissected and stored in 70% ethanol. The head was imaged using an lCT40 Scanco vivaCT42 (Scanco Medical, Bassersdorf, Switzerland), with the following settings: integration time, 300 ms; 45 E(kVp); 177 mA. Three-dimensional images were reconstructed using OsiriX software (64-bit version; Pixmeo, Bernex, Switzerland). Femurs were imaged using a Skyscan 1272 system (Bruker, USA), with the following scanning parameters: image pixel size, 5 µm; X-ray voltage, 60 kV; X-ray current, 166 µA; filter, A1 0.71 mm; exposure, 1800 ms; rotation step, 0.71°; frame averaging, 3; tomographic rotation, 180°. Cross-sections were reconstructed using NRecon software (Bruker), with the following parameters: smoothing, 0; ring artefact reduction, 2; beam-hardening correction, 40%. The Dataviewer, CTAn and CTVox software packages (Skyscan) were used to visualize and determine bone histomorphometric variables. For each variable, the volume of interest was proportional to the length of the femur. Cortical bone was analyzed in the midshaft of the femur. The volume of interest ranged from 5% above the midshaft to 5% below. Trabecular bone was analyzed at the distal femoral metaphysis. The region of interest was delimited manually between the end of the primary spongiosa and a site 15% lower.

### Isolation of osteoblastic cells from calvaria

Primary OBs from calvaria of 2- to 5-day-old *Osx-Fgfr3*, *Col1-Fgfr3* and control mice were isolated and cultured as described previously (Mugniery et al., 2012). The cells were obtained by digestion with collagenase IV (C5138, Sigma-Aldrich, Saint Louis, MO, USA), expanded in α-MEM medium (Gibco, Carlsbad, CA, USA) complemented with 10% fetal calf serum, 2 mM glutamine, 100 U/ml penicillin and 100 mg/ml streptomycin, and cultured in a humidified 5% CO_2_ atmosphere at 37°C. At subconfluence, OBs were harvested with trypsin/EDTA (Invitrogen, Carlsbad, CA, USA).

### *In vitro* OB proliferation, differentiation and mineralization assays

For proliferation studies, harvested cells were plated in 96-well plates at a density of 1000 cells/well. Proliferation over 7 days was measured using the IncuCyte live-content imaging platform (Sartorius). Images were acquired every 3 h with an X4 lens. For differentiation and mineralization studies, we used differentiation medium [50 µM ascorbic acid and 10 mM β-glycerophosphate disodium salt hydrate (Sigma-Aldrich)]. Cells were plated at a density of 12,500/cm². Osteogenic differentiation was assessed on day 7, when cells were stained for ALP activity using bromochloroindoyl-phosphate/nitroblue tetrazolium chloride (Sigma-Aldrich). Mineralization was detected on day 21, using Alizarin Red staining (Sigma-Aldrich).

### Statistics

Differences between experimental groups were assessed with an analysis of variance or the Mann–Whitney test. The threshold for statistical significance was set to *P*≤0.05. All statistical analyses were performed using Prism software (version 5; GraphPad Software, La Jolla, CA, USA). All values were quoted as the mean±s.d.
